# The Effect of Microneedling Mesotherapy, Chemical Exfoliation and Combination Therapy on Selected Skin Parameters and *Cutibacterium acnes* Colonization in Women With Oily Skin

**DOI:** 10.1111/jocd.70752

**Published:** 2026-02-19

**Authors:** Agnieszka Ciozda, Ewelina Firlej, Joanna Bartosińska, Dorota Raczkiewicz

**Affiliations:** ^1^ Doctoral School Medical University of Lublin Lublin Poland; ^2^ Department of Cosmetology and Aesthetic Medicine Medical University of Lublin Lublin Poland; ^3^ Department of Health Economics, Management, and Statistics, School of Public Health Centre of Postgraduate Medical Education Warsaw Poland

**Keywords:** bacterial activity, porphyrins, seborrhoeic skin, skin parameters

## Abstract

**Background:**

Oily skin is characterized by excessive sebum production and increased activity of *Cutibacterium acnes*.

**Aims:**

To evaluate the effects of mandelic acid exfoliation, microneedle mesotherapy with a sebum‐regulating ampoule, and combination therapy on skin parameters and *C. acnes* activity in women with oily skin.

**Methods:**

Fifty‐five women aged 18–47 were randomly assigned to three groups: microneedle mesotherapy (6 treatments), mandelic acid exfoliation (6 treatments), or combination therapy (3 exfoliations followed by 3 mesotherapy treatments). Skin hydration, sebum level, pH, and gloss were measured using an MPA Courage&Khazaka device. *C. acnes* activity was assessed indirectly by porphyrin fluorescence with a Visiopor device. Measurements were taken on the cheeks and in the T‐zone before treatment (day 1), after three session (day 63), and after six sessions (day 126).

**Results:**

Skin hydration increased significantly in all groups. Mesotherapy reduced sebum in the T‐zone only after six treatments, while exfoliation reduced sebum mainly on the cheeks. Combination therapy resulted in the most extensive and stable sebum reduction in both areas. A temporary decrease in pH was observed after mesotherapy, with no pH changes after exfoliation or combination therapy. No significant changes in skin gloss were found. *C. acnes* colonization increased in the T‐zone after mesotherapy, whereas exfoliation and combination therapy significantly reduced all colonization indices, with the strongest effect for combination therapy.

**Conclusions:**

Mandelic acid exfoliation and microneedle mesotherapy support regulation of sebaceous activity and epidermal barrier function. Combination therapy is therapeutically superior, providing optimal improvement in skin parameters and the most effective reduction of *C. acnes* activity.

**Abbreviations**
AHA: alpha hydroxy acids
*C. acnes*
: *Cutibacterium acnes*
*F*
: ANOVA *F* statistic
H: Kruskal–Wallis test statistic
*M*
: mean
Max: maximum value
Me: median
Min: minimum value
*N*
: number of participants
*p*
: *p*—significance level (*p*‐value)
PDGF: platelet‐derived growth factor
post hoc: results of post hoc multiple comparisons
SD: standard deviation
S‐W: Shapiro–Wilk test statistic
TGF‐β: transforming growth factor β
UVRF: UV‐induced red fluorescence
VEGF: vascular endothelial growth factor

## 1 Introduction

Oily skin is one of the most common dermatological problems. It is characterized by increased activity of the sebaceous glands, leading to excessive sebum production and impaired epidermal barrier function. As a result, the hydrolipid layer is damaged and changes in the composition of the skin microbiome occur, including an increase in the number of *Cutibacterium acnes* bacteria. Their ability to form biofilm plays an important role in the pathogenesis of acne and seborrhoea [1, 2, 3, 4]. Advances in cosmetology and aesthetic dermatology have made it possible to use minimally invasive procedures that affect both the physiology of the skin and its microenvironment. Among them, microneedle mesotherapy and chemical exfoliation are of particular importance, as they influence skin parameters, support its regeneration, and affect the composition of its microbiome.

Microneedling involves controlled mechanical damage to the skin using thin needles, which initiates a series of regenerative processes, such as fibroblast activation, neocollagenesis, extracellular matrix remodeling and increased permeability of the stratum corneum [5, 6]. The resulting micro‐injuries stimulate the release of growth factors: platelet‐derived (PDGF), transforming (TGF‐β) and vascular endothelial (VEGF), which leads to thickening of the dermis, improvement of its elasticity and microcirculation [7]. Clinical studies confirm that microneedling is effective not only in skin rejuvenation, but also in the treatment of seborrhoeic skin. The treatment improves the skin structure by narrowing enlarged pores and increasing the penetration of active substances applied topically during the procedure [8, 9]. Microneedle mesotherapy causes a controlled, temporary inflammatory reaction in the skin, which leads to increased synthesis of collagen, elastin, glycosaminoglycans and keratinocyte proliferation [10]. Recent reports indicate that microneedling can also modulate the skin microbiome by mechanically removing *C. acnes* biofilm and increasing oxygenation of hair follicle openings, which limits the proliferation of anaerobic bacteria [11]. The treatment is characterized by a high safety profile, a short recovery period and a low risk of complications, provided that the depth of the microneedle punctures is correctly selected [12, 13].

Chemical exfoliation is a procedure involving controlled exfoliation of the skin using keratolytic substances such as alpha, beta and polyhydroxy acids. The mechanism of action involves loosening the connections between corneocytes in the stratum corneum, removing excess keratinised cells, normalizing keratinisation processes and stimulating cell renewal [14]. Chemical peels containing mandelic acid, gluconolactone or salicylic acid are widely used in the treatment of oily and seborrhoeic skin, as they reduce sebum secretion, unclog sebaceous gland openings and improve skin texture by narrowing enlarged pores [15, 16, 17]. In addition to its keratolytic effect, chemical exfoliation supports cell renewal and improves the functioning of the epidermal barrier by accelerating the regeneration of the stratum corneum and improving the structure and lipid cohesion of the epidermal barrier [18]. Regular use of chemical peels can also indirectly affect the skin's microenvironment by reducing *C. acnes* biofilm and normalizing pH, which promotes microbial balance [19, 20]. In the case of oily skin, this treatment is not only aesthetic but also therapeutic, combining exfoliating, sebum‐regulating, and epidermal barrier–enhancing effects.

Combining chemical exfoliation with microneedle mesotherapy is an increasingly common clinical practice based on the principle of synergy. Exfoliating the stratum corneum before microneedling increases its permeability and allows for deeper penetration of active substances, which can enhance the therapeutic effect [18]. Studies indicate that the sequential use of these treatments leads to a more comprehensive improvement in skin parameters: increased hydration, normalized sebum secretion, and stabilization of the microbiome compared to the use of a single procedure [8, 21]. However, there is still a lack of comparative studies analyzing the three therapeutic approaches: monotherapy with microneedle mesotherapy, monotherapy with chemical exfoliation, and combination therapy, in the context of changes in the biochemical and microbiological parameters of oily skin. This study is an attempt to fill this gap.

## 2 Materials and Methods

### 2.1 Study Design and Participants

The aim of the study was to evaluate the effect of microneedle mesotherapy, mandelic acid exfoliation, and combination therapy on selected skin parameters in women with oily skin. The study was conducted between January 2023 and May 2025. All procedures were performed in accordance with the principles of the Declaration of Helsinki, and the study protocol was approved by the Bioethics Committee. Each participant was informed in detail about the purpose and course of the study and gave her written informed consent to participate in the study.

The study involved 55 women aged 18 to 47 who met the inclusion criteria. The participants were randomly assigned to one of three independent groups, differing in the type of therapy used. There were no statistically significant differences between the treatment groups regarding age or the duration of seborrhea. The duration of the study was 126 days (18 weeks).

Group I (microneedling mesotherapy) included 19 women who underwent a series of six microneedling mesotherapy treatments using a sebum‐regulating ampoule (Dives Med, Acneout, Germany).

Group II (mandelic acid exfoliation) included 18 women who underwent a series of six exfoliation treatments using a mandelic acid preparation (Fenice, Mandel X, Italy).

Group III (combination therapy) included 18 women who underwent three mandelic acid exfoliation treatments (Fenice, Mandel X, Italy) followed by three microneedle mesotherapy treatments using a sebum‐regulating ampoule (Dives Med, Acneout, Germany).

Detailed data on the age of participants and the duration of excessive sebum secretion in individual groups are presented in Table 1.

**TABLE 1:** Characteristics of the treatment groups.

	Min	Max	*M*	Me	SD
Treatment group 1 (*N* = 19)					
Age	18	42	27.89	27.00	7.24
Duration	5	28	11.84	10.00	6.69
Treatment group 2 (*N* = 18)					
Age	19	46	25.22	23.00	6.50
Duration	1	30	9.56	8.00	6.73
Treatment group 3 (*N* = 18)					
Age	18	47	28.11	26.00	8.64
Duration	3	20	10.33	10.00	5.25

All participants fulfilled the inclusion criteria for the study, which were: age ≥ 18 years, presence of oily skin, and provision of written informed consent to participate in the study. The exclusion criteria for the study were: active fungal, bacterial, or viral skin infections; fever; recent sunburn or artificial tanning; inflammatory stage of acne; wounds; pregnancy and breastfeeding; tendency to form keloids; allergy to nickel (used in needles); coagulation disorders; neoplastic, metabolic or autoimmune diseases; and hypersensitivity to ingredients of the applied preparations.

### 2.2 Treatment Protocols

#### 2.2.1 Microneedle Mesotherapy Protocol

The microneedle mesotherapy treatment was performed using a pen‐type device (m.pen [pro], Mesoestetic). The needle penetration depth was precisely adjusted according to the treatment area: 0.25 mm for the forehead, nose, chin, and under‐eye area, and 0.5 mm for the cheeks. Microneedling was performed using the stamping technique. The device operated at a speed of 9500 punctures per minute, utilizing cartridges containing 24 needles. Microneedle mesotherapy treatments were performed at intervals of 21 days (6 treatments in group I and 3 treatments in group III). The procedure was performed using a cosmetic formulation with sebum‐regulating effects (Dives Med, Acneout, Germany), containing the following active substances:

- Biomimetic peptide: Myristoyl Hexapeptide‐23.
- Non‐crosslinked hyaluronic acid: 5 mg/mL.
- Retinol.
- Vitamins: Tocopherol (vitamin E), pyridoxine (vitamin B6), panthenol (pro‐vitamin B5), cyanocobalamin (vitamin B12).
- Strengthening agents: Zinc, copper pyrrolidine carboxylate, glycolic acid, allantoin.

The choice of preparation used in microneedle mesotherapy was based on its synergistic effect supporting the functions of oily skin. The biomimetic peptide Myristoyl Hexapeptide‐23 has an antibacterial effect against *C. acnes*. Allantoin has well‐documented soothing and calming properties. Non‐cross‐linked hyaluronic acid provides short‐term but effective skin hydration without a heavy feeling. Retinol and a complex of vitamins (E, B6, B5, B12) regulate the keratinisation process, support the barrier functions of the epidermis, and provide antioxidant effects. Zinc and copper PCA, known for their anti‐inflammatory and sebum‐normalizing properties, have been selected as ingredients that support the balance of seborrhoeic skin. Glycolic acid, as an alpha hydroxy acid with keratolytic properties, promotes cell renewal and improves the appearance of oily skin.

#### 2.2.2 Exfoliation Protocol

Exfoliation was performed using a surface‐acting preparation (Fenice, Mandel X, Italy) with a pH < 1, containing 60% mandelic acid enriched with citric acid, quercetin, and acetic acid. The preparation was applied to cleansed and defatted skin and then neutralized with water after 20–30 min, in accordance with the manufacturer's instructions. Exfoliation treatments were performed at intervals of 21 days (6 treatments in group II and 3 treatments in group III). The choice of exfoliation preparation was based on the properties of mandelic acid, which acts on the surface, is well tolerated even by people with sensitive and seborrhoeic skin, and has the ability to reduce microbial colonization. The addition of citric acid, quercetin, and acetic acid increases the keratolytic and antioxidant effect of the preparation and promotes even exfoliation of the epidermis.

### 2.3 Skin Parameter Measurements

Skin parameters were measured three times: on the day of enrolment in the study (day 1), after the third treatment (day 63), and after the sixth treatment (day 126). The analysis was performed using the multi‐module MPA system (Courage + Khazaka Electronic GmbH, Cologne, Germany) equipped with the following measuring probes:

- Corneometer CM 825—a probe for assessing the hydration of the stratum corneum, using electrical capacitance measurement as an indicator of water content. The results were presented in arbitrary units (AU), where higher values indicate greater hydration of the stratum corneum.
- Sebumeter SM 815—a probe used for quantitative assessment of sebum levels by measuring the amount of surface lipids. The results were expressed in μg/cm^2^, with higher values corresponding to greater sebum production.
- pH‐Meter—a probe used to measure skin pH. The result was given in pH units, according to the standard 0–14 scale. Changes in pH reflect the functioning of the hydrolipid barrier.
- Glossymeter GL 200—a probe used to assess skin gloss by measuring light reflection from its surface. The parameter measured is Gloss DSC (Differential Specular Component), which determines the amount of mirror‐like light reflected, separating it from diffuse reflection. The results are given in Gloss Units (GU), with higher values corresponding to greater skin gloss, which may reflect its smoothness, hydration, and the presence of surface lipids.

In addition, a Visiopor PP 34 N device (Courage + Khazaka Electronic GmbH, Cologne, Germany) was used to analyze *C. acnes* colonization of the skin based on fluorescence. The system utilizes the orange‐red fluorescence of porphyrins produced by *C. acnes* under the influence of UV‐A light. The analysis included three indicators:

- Percentage of area with fluorescence—the percentage of the examined skin area exhibiting fluorescence. The results were expressed as a percentage (%).
- Number of follicles with fluorescence—number of hair follicles showing fluorescence, unit: number of pores (count).
- Average fluorescence intensity—average fluorescence intensity in the examined area, which is an indirect indicator of metabolic activity and increased porphyrin production by *C. acnes*. The results were expressed in arbitrary fluorescence units (AU).

Higher values of all Visiopor parameters indicate increased colonization of the skin by *C. acnes* and greater bacterial activity.

Before the start of the study, all measuring probes were calibrated in accordance with the manufacturer's instructions to ensure the reliability and repeatability of the results. Measurements were taken in two areas: the T‐zone (forehead, nose, chin) and the cheeks, with the measuring points remaining in a fixed location. All measurements were performed in a room with controlled environmental conditions (temperature 21°C–23°C, relative humidity 45%–55%). Participants remained at rest for 30 min before the measurements began, which ensured the stabilization of skin parameters.

### 2.4 Statistical Analysis

Statistical analysis was performed using IBM SPSS Statistics, Version 26.0 (IBM Corp., Armonk, NY, USA). In the first stage, the distribution of continuous variables was assessed using the Shapiro–Wilk test, which allowed for verification of compliance with normal distribution and selection of appropriate analytical methods. Due to the fact that most of the variables studied did not meet the criterion of normal distribution, non‐parametric methods were used in further analysis.

For each study group, Friedman's test (Friedman's ANOVA) was performed to assess the differences between the three measurement points (pre‐treatment, after three treatments, and after six treatments). When the Friedman test showed statistical significance, multiple comparisons were performed to determine between which measurements there were significant differences. The results of these analyses are presented in tables in the form of post hoc designations, indicating the direction of change.

Comparisons between the three treatment groups (microneedle mesotherapy, mandelic acid exfoliation, combined therapy) were performed using the Kruskal–Wallis test. If this test indicated statistical significance, multiple comparisons were performed to determine which groups differed statistically significantly from each other. The results are presented in the tables in the same way—using post hoc tests with an indication of the direction of the differences.

Descriptive data were presented as: arithmetic mean (M), median (Me), standard deviation (SD), minimum (Min) and maximum (Max) values. Three levels of statistical significance were adopted: *p* < 0.05 (*), *p* < 0.01 (**) and *p* < 0.001 (***). Results were considered statistically significant at *p* < 0.05.

## 3 Results

### 3.1 Skin Parameter Measurement Results

#### 3.1.1 Treatment Group I (Microneedle Mesotherapy)

Friedman's ANOVA showed statistically significant changes in hydration levels in both the T‐zone and cheeks. The hydration level in the T‐zone increased significantly after 6 treatments compared to the condition before and after 3 treatments (*p* < 0.001), while the hydration level in the cheeks increased significantly after 3 treatments and after 6 treatments compared to the condition before the treatments (*p* < 0.001).

Sebum levels in the T‐zone decreased significantly after six treatments compared to after three treatments and before the treatments (*p* < 0.001). Sebum levels in the cheeks decreased significantly after three treatments and remained reduced after six treatments (*p* < 0.001).

Friedman's ANOVA showed statistically significant changes in pH levels in both the T‐zone and cheeks. The pH level of the T‐zone and cheeks after 3 treatments decreased significantly (*p* = 0.021).

No statistically significant changes in gloss were found after 3 and 6 mesotherapy treatments in either the T‐zone or the cheeks.

Fluorescence imaging analysis of the skin showed a statistically significant increase in the number of fluorescent follicles after the third treatment, which persisted after the completion of the full series of six treatments (*p* = 0.006). In addition, the average fluorescence intensity in the T‐zone and on the cheeks decreased significantly after six treatments compared to both the baseline and after three treatments (*p* = 0.002; 0.001, respectively). Detailed measurement results are presented in Table 2.

**TABLE 2:** Comparison of hydration levels, sebum levels, pH, skin gloss, and *Cutibacterium acnes* colonization before treatment, after three sessions, and after six microneedle mesotherapy treatments.

Skin parameter	Measurement area	Measurement	Min	Max	*M*	Me	SD	*F*	*p*	Post hoc
Hydration (AU)	T‐zone	Day 1 (1)	24.53	59.38	43.88	44.28	8.01	19.263	< 0.001***	3 > 2,1
		Day 63 (2)	35.93	70.85	49.15	47.68	8.31			
		Day 126 (3)	44.38	64.15	54.01	54.33	5.35			
	Cheeks	Day 1 (1)	20.78	53.05	35.93	33.88	8.29	23.707	< 0.001***	2,3 > 1
		Day 63 (2)	42.98	64.50	53.34	53.13	6.18			
		Day 126 (3)	46.73	64.70	55.61	55.43	4.83			
Sebum level (μg/cm^2^)	T‐zone	Day 1 (1)	137.50	248.75	192.22	188.00	30.58	24.947	< 0.001***	3 < 1,2
		Day 63 (2)	116.25	238.00	170.21	176.25	30.42			
		Day 126 (3)	66.25	176.75	127.72	128.50	27.22			
	Cheeks	Day 1 (1)	114.75	251.25	185.00	191.00	36.22	20.947	< 0.001***	1 > 2,3
		Day 63 (2)	88.75	197.50	147.61	149.50	36.29			
		Day 126 (3)	55.00	163.00	114.84	115.25	30.07			
pH	T‐zone	Day 1 (1)	5.50	8.12	6.33	6.23	0.64	7.684	0.021*	1 > 2
		Day 63 (2)	5.53	6.68	5.90	5.81	0.31			
		Day 126 (3)	5.67	6.38	6.01	5.99	0.17			
	Cheeks	Day 1 (1)	5.23	7.27	6.14	6.12	0.50	7.895	0.021*	2 < 1,3
		Day 63 (2)	5.31	7.06	5.84	5.78	0.42			
		Day 126 (3)	5.67	6.31	6.03	6.02	0.17			
Gloss (DSC) (GU)	T‐zone	Day 1 (1)	3.00	8.54	5.46	5.26	1.25	1.263	0.532	—
		Day 63 (2)	3.12	9.69	5.29	5.11	1.43			
		Day 126 (3)	3.58	7.64	5.60	5.90	1.16			
	Cheeks	Day 1 (1)	3.22	10.82	5.29	5.45	1.76	3.263	0.196	—
		Day 63 (2)	2.61	8.42	4.64	4.34	1.32			
		Day 126 (3)	2.62	6.95	4.87	4.66	1.20			
Percentage of area with fluorescence (%)	T zone	Day 1 (1)	0.21	5.96	2.98	2.93	1.75	2.947	0.229	—
		Day 63 (2)	0.26	7.26	3.73	3.70	2.04			
		Day 126 (3)	0.38	7.57	3.45	3.37	1.85			
	Cheeks	Day 1 (1)	0.51	4.85	2.44	2.09	1.50	4.105	0.128	—
		Day 63 (2)	0.43	7.82	3.45	3.64	2.17			
		Day 126 (3)	0.40	7.32	3.37	2.83	2.31			
Number of follicles with fluorescence (count)	T zone	Day 1 (1)	6.00	56.50	32.76	35.75	13.08	10.211	0.006**	1 < 2,3
		Day 63 (2)	6.75	71.25	41.11	36.00	19.24			
		Day 126 (3)	7.50	77.00	43.32	50.00	18.42			
	Cheeks	Day 1 (1)	9.00	87.75	34.33	31.00	19.46	0.737	0.692	—
		Day 63 (2)	9.50	114.50	39.89	30.00	26.32			
		Day 126 (3)	5.50	93.25	40.09	44.00	22.21			
Average fluorescence intensity (AU)	T zone	Day 1 (1)	228.25	251.75	243.20	246.50	7.94	12.827	0.002**	3 < 2,1
		Day 63 (2)	175.75	252.00	237.54	242.50	18.13			
		Day 126 (3)	185.75	250.25	232.66	237.25	15.48			
	Cheeks	Day 1 (1)	178.75	252.50	238.46	243.25	17.25	13.053	0.001**	3 < 2,1
		Day 63 (2)	208.75	251.75	237.14	241.25	11.59			
		Day 126 (3)	176.25	250.00	226.01	226.75	19.99			

* *p* < 0.05.

** *p* < 0.01.

*** *p* < 0.001.

#### 3.1.2 Treatment Group 2 (Exfoliation)

Friedman's ANOVA showed statistically significant changes in hydration levels in both the T‐zone and cheeks. The hydration level in the T‐zone increased significantly after 6 treatments compared to the condition before and after 3 treatments (*p* = 0.002), while the hydration level in the cheeks increased significantly after 6 treatments compared to the condition before the treatments (*p* = 0.006).

Friedman's ANOVA revealed statistically significant changes in cheek sebum levels, with a decrease observed after six treatments compared to baseline and after three treatments (*p* = 0.009).

No statistically significant changes in pH and gloss were found after 3 and 6 exfoliation treatments, either in the T‐zone or on the cheeks.

Statistically significant changes in the percentage of fluorescent area were demonstrated. In the T‐zone, a significant increase in this parameter was observed after three treatments, while after six treatments, this value decreased, reaching a level similar to the initial state (*p* = 0.024). On the cheeks, the percentage of fluorescent area decreased significantly after six treatments compared to the pre‐treatment state (*p* = 0.004). A significant reduction in the average fluorescence intensity was demonstrated in both areas studied, the T‐zone and cheeks, after six treatments compared to the pre‐treatment state and after the third treatment (*p* < 0.001). Table 3 illustrates the data in detail.

**TABLE 3:** Comparison of hydration levels, sebum levels, pH, skin gloss, and *Cutibacterium acnes* colonization before treatment, after three sessions, and after six exfoliation treatments.

Skin parameter	Measurement area	Measurement	Min	Max	*M*	Me	SD	*F*	*p*	Post hoc
Hydration (AU)	T‐zone	Day 1 (1)	34.50	49.50	42.98	43.03	4.29	12.111	0.002**	3 > 2,1
		Day 63 (2)	36.28	54.25	44.16	43.04	5.80			
		Day 126 (3)	40.83	56.88	48.62	48.79	4.82			
	Cheeks	Day 1 (1)	27.45	53.63	43.44	45.63	7.06	10.111	0.006**	3 > 1
		Day 63 (2)	34.93	61.85	47.73	45.75	7.62			
		Day 126 (3)	44.78	63.05	51.66	52.28	4.82			
Sebum level (μg/cm^2^)	T‐zone	Day 1 (1)	143.25	245.25	192.17	192.50	27.07	4.111	0.128	—
		Day 63 (2)	43.50	359.75	196.11	190.00	77.25			
		Day 126 (3)	32.00	246.50	156.28	146.50	57.89			
	Cheeks	Day 1 (1)	89.50	243.75	188.44	189.63	40.93	9.333	0.009**	1,2 > 3
		Day 63 (2)	31.75	437.25	188.74	168.13	97.35			
		Day 126 (3)	27.25	228.25	140.54	147.50	50.32			
pH	T‐zone	Day 1 (1)	5.44	7.75	6.16	5.89	0.61	3.111	0.211	—
		Day 63 (2)	5.60	6.36	5.82	5.78	0.19			
		Day 126 (3)	5.48	6.08	5.81	5.77	0.19			
	Cheeks	Day 1 (1)	5.32	6.79	5.93	5.71	0.51	0.333	0.846	—
		Day 63 (2)	5.44	6.63	5.88	5.88	0.29			
		Day 126 (3)	5.34	6.20	5.75	5.76	0.19			
Gloss (DSC) (GU)	T‐zone	Day 1 (1)	2.24	8.03	5.49	5.79	1.45	3.296	0.192	—
		Day 63 (2)	2.02	9.04	4.92	4.51	1.58			
		Day 126 (3)	2.24	8.79	5.35	5.48	1.78			
	Cheeks	Day 1 (1)	2.88	9.33	5.21	4.61	1.74	5.333	0.069	—
		Day 63 (2)	2.66	9.78	5.54	5.57	1.85			
		Day 126 (3)	2.16	8.76	4.77	4.49	1.54			
Percentage of area with fluorescence (%)	T zone	Day 1 (1)	0.25	6.67	3.78	4.02	2.10	7.444	0.024*	2 > 3
		Day 63 (2)	0.01	6.42	4.04	4.42	1.77			
		Day 126 (3)	0.05	8.17	3.30	3.44	2.33			
	Cheeks	Day 1 (1)	0.00	7.83	3.92	4.04	2.10	10.491	0.004**	1,2 > 3
		Day 63 (2)	0.00	6.80	3.71	3.81	1.86			
		Day 126 (3)	0.00	8.53	2.73	2.34	2.03			
Number of follicles with fluorescence (count)	T zone	Day 1 (1)	4.50	79.50	46.17	49.25	21.45	1.400	0.497	—
		Day 63 (2)	0.25	104.25	53.67	55.63	24.64			
		Day 126 (3)	2.75	86.75	46.72	48.50	27.98			
	Cheeks	Day 1 (1)	0.00	113.00	49.50	50.38	25.84	4.588	0.101	—
		Day 63 (2)	0.00	91.75	48.57	53.25	22.91			
		Day 126 (3)	0.00	89.25	40.26	38.63	23.97			
Average fluorescence intensity (AU)	T zone	Day 1 (1)	173.00	252.75	234.12	242.75	21.02	29.778	< 0.001***	1,2 > 3
		Day 63 (2)	50.25	251.50	210.44	227.88	49.21			
		Day 126 (3)	42.00	215.75	169.74	185.00	40.21			
	Cheeks	Day 1 (1)	0.00	250.50	223.17	239.25	57.75	21.939	< 0.001***	1,2 > 3
		Day 63 (2)	0.00	250.50	202.50	230.13	65.58			
		Day 126 (3)	0.00	226.75	170.85	187.88	48.72			

* *p* < 0.05.

** *p* < 0.01.

*** *p* < 0.001.

#### 3.1.3 Treatment Group III (Combination Therapy)

Friedman's ANOVA showed statistically significant changes in hydration levels in both the T‐zone and cheeks. Both the hydration levels of the T‐zone and cheeks increased significantly after 3 treatments compared to the condition before the treatments and remained stable after 6 treatments (*p* = 0.001; < 0.001, respectively).

As shown by Friedman's ANOVA, the level of sebum on both the cheeks and the T‐zone after 6 treatments decreased significantly compared to the condition after 3 treatments and before the treatments (*p* < 0.001).

No statistically significant changes in pH and gloss were found after 3 and 6 combined treatments in either the T‐zone or the cheeks.

The analysis showed a significant decrease in the percentage of fluorescent area in both the T‐zone and cheeks after six treatments compared to baseline and after three treatments (*p* = 0.034; 0.001, respectively). Regarding the number of follicles with fluorescence on the cheeks, a significant reduction was observed after six treatments compared to baseline values (*p* = 0.017). For the average fluorescence intensity in the T‐zone, a statistically significant decrease was observed after three treatments compared to baseline, as well as after six treatments compared to both baseline and post‐three‐treatment values (*p* < 0.001). In the cheeks, a significant decrease in average fluorescence intensity was observed after six treatments compared to both baseline and the measurement after the third treatment (*p* < 0.001). Table 4 contains detailed data.

**TABLE 4:** Comparison of hydration levels, sebum levels, pH, skin gloss, and *Cutibacterium acnes* colonization before treatment, after three sessions, and after six combination treatments.

Skin parameter	Measurement area	Measurement	Min	Max	*M*	Me	SD	*F*	*p*	Post hoc
Hydration (AU)	T‐zone	Day 1 (1)	30.18	59.00	45.10	44.75	8.04	14.778	0.001**	3,2 > 1
		Day 63 (2)	39.70	59.38	50.21	51.00	6.39			
		Day 126 (3)	42.65	70.73	52.57	52.97	6.39			
	Cheeks	Day 1 (1)	20.70	64.83	42.89	43.02	11.64	19.000	< 0.001***	3,2 > 1
		Day 63 (2)	39.00	62.43	53.10	54.07	6.68			
		Day 126 (3)	45.05	72.63	55.73	56.31	6.62			
Sebum level (μg/cm^2^)	T‐zone	Day 1 (1)	165.50	262.00	200.94	185.38	27.90	21.444	< 0.001***	1,2 > 3
		Day 63 (2)	111.25	263.50	193.97	196.00	48.94			
		Day 126 (3)	58.75	174.00	133.11	151.25	35.15			
	Cheeks	Day 1 (1)	95.00	251.75	169.75	159.63	58.59	16.444	< 0.001***	1,2 > 3
		Day 63 (2)	67.75	269.75	179.65	189.38	61.98			
		Day 126 (3)	59.75	161.50	108.08	111.25	34.05			
pH	T‐zone	Day 1 (1)	4.06	8.32	5.88	5.75	1.04	4.778	0.092	—
		Day 63 (2)	5.28	6.84	5.91	5.85	0.40			
		Day 126 (3)	5.55	6.19	5.91	5.91	0.21			
	Cheeks	Day 1 (1)	4.93	7.06	5.86	5.82	0.54	3.111	0.211	—
		Day 63 (2)	5.26	7.52	5.90	5.72	0.57			
		Day 126 (3)	5.52	6.20	5.96	5.98	0.19			
Gloss (DSC) (GU)	T‐zone	Day 1 (1)	3.52	8.19	5.31	5.11	1.31	1.444	0.486	—
		Day 63 (2)	3.08	8.79	5.38	5.28	1.52			
		Day 126 (3)	2.84	8.50	5.21	5.47	1.52			
	Cheeks	Day 1 (1)	3.14	7.98	5.63	5.49	1.59	1.000	0.607	—
		Day 63 (2)	3.44	8.67	5.73	5.61	1.63			
		Day 126 (3)	2.24	9.28	5.16	5.06	1.78			
Percentage of area with fluorescence (%)	T zone	Day 1 (1)	1.11	5.95	3.25	3.33	1.52	6.778	0.034*	1,2 > 3
		Day 63 (2)	0.06	8.94	3.24	3.43	2.08			
		Day 126 (3)	0.13	6.03	2.31	2.47	1.63			
	Cheeks	Day 1 (1)	0.19	4.34	2.58	2.70	1.13	14.333	0.001**	1,2 > 3
		Day 63 (2)	0.06	6.53	2.42	2.28	1.61			
		Day 126 (3)	0.09	5.06	1.70	1.16	1.40			
Number of follicles with fluorescence (count)	T zone	Day 1 (1)	10.75	70.75	33.22	32.50	17.93	5.600	0.061	—
		Day 63 (2)	1.25	68.00	33.61	33.88	19.03			
		Day 126 (3)	2.50	64.75	30.00	34.50	19.00			
	Cheeks	Day 1 (1)	3.75	60.50	29.85	29.00	14.28	8.111	0.017*	1 > 3
		Day 63 (2)	1.75	77.00	27.61	27.38	17.81			
		Day 126 (3)	1.50	53.25	22.94	18.25	15.04			
Average fluorescence intensity (AU)	T zone	Day 1 (1)	211.00	250.50	237.64	239.38	10.45	28.444	< 0.001***	1 > 2 > 3
		Day 63 (2)	151.75	251.50	217.37	232.88	31.79			
		Day 126 (3)	65.50	235.75	190.58	198.75	41.65			
	Cheeks	Day 1 (1)	162.00	250.25	232.01	236.63	20.84	24.111	< 0.001***	1,2 > 3
		Day 63 (2)	108.00	250.50	216.06	233.63	39.37			
		Day 126 (3)	91.00	240.50	182.29	181.50	45.28			

* *p* < 0.05.

** *p* < 0.01.

*** *p* < 0.001.

### 3.2 Comparison of Three Treatment Procedures

In order to compare the effectiveness of the three procedures used—microneedle mesotherapy, exfoliation and a combination treatment—a statistical analysis was performed using the Kruskal‐Wallis test. The effect size was determined as the difference between the values obtained after six treatments and the baseline values before the start of therapy.

The analysis showed that the treatments did not differ significantly in terms of changes in hydration levels in the T‐zone, while in the case of the cheeks, a significantly greater increase in hydration was observed after mesotherapy compared to exfoliation. With regard to changes in skin pH, no significant differences were found between the procedures tested, both in the T‐zone and on the cheeks. Similarly, no statistically significant differences were observed between the treatments in terms of changes in skin sebum and gloss levels in both the T‐zone and cheeks. Detailed data are presented in Table 5.

**TABLE 5:** Comparison of treatments in terms of the effect measured by changes in hydration, sebum, pH, and gloss levels.

Skin parameter	Measurement area	Treatment group	Min	Max	*M*	Me	SD	H	*p*	Post hoc
Hydration	T‐zone	Microneedling (1)	−3.80	24.40	10.13	8.20	7.08	5.089	0.079	—
		Exfoliation (2)	−0.38	16.62	5.65	3.36	5.77			
		Combined treatment (3)	−10.17	23.73	7.47	8.05	8.62			
	Cheeks	Microneedling (1)	2.38	34.40	19.68	18.27	9.65	11.863	0.003**	1 > 2
		Exfoliation (2)	−0.70	19.88	8.22	6.71	6.14			
		Combined treatment (3)	−11.25	28.25	12.84	13.22	10.80			
Sebum level	T‐zone	Microneedling (1)	−141.75	−12.00	−64.50	−57.50	35.27	4.850	0.088	—
		Exfoliation (2)	−118.50	49.00	−35.89	−34.37	51.06			
		Combined treatment (3)	−124.00	−2.25	−67.83	−64.75	38.31			
	Cheeks	Microneedling (1)	−174.50	22.50	−70.16	−71.00	49.55	2.069	0.355	—
		Exfoliation (2)	−154.00	49.50	−47.90	−42.00	52.49			
		Combined treatment (3)	−146.50	15.75	−61.67	−54.00	47.32			
pH	T‐zone	Microneedling (1)	−2.17	0.58	−0.32	−0.24	0.72	5.935	0.051	—
		Exfoliation (2)	−1.98	0.55	−0.34	−0.20	0.66			
		Combined treatment (3)	−2.13	1.70	0.03	0.28	0.95			
	Cheeks	Microneedling (1)	−1.30	0.93	−0.11	−0.18	0.54	2.069	0.355	—
		Exfoliation (2)	−1.15	0.67	−0.18	0.04	0.55			
		Combined treatment (3)	−0.91	1.05	0.10	0.14	0.52			
Gloss (DSC)	T‐zone	Microneedling (1)	−2.39	1.45	0.14	0.37	1.05	1.127	0.569	—
		Exfoliation (2)	−3.02	1.54	−0.14	−0.01	1.15			
		Combined treatment (3)	−2.35	2.84	−0.11	−0.21	1.32			
	Cheeks	Microneedling (1)	−4.38	2.72	−0.42	−0.58	1.48	0.021	0.990	—
		Exfoliation (2)	−3.22	1.53	−0.45	0.13	1.31			
		Combined treatment (3)	−2.46	1.30	−0.46	−0.56	1.23			

** *p* < 0.01.

The most pronounced differences between the three procedures were observed in relation to the level of *C. acnes* colonization. In the case of microneedle mesotherapy, a slight increase in colonization was observed, while after exfoliation and the combined treatment, there was a marked decrease in this parameter. A similar trend was observed in the number of fluorescent follicles—microneedle mesotherapy caused an increase, while exfoliation and the combined treatment led to a decrease. In addition, the average fluorescence intensity decreased significantly after exfoliation and the combined treatment, while the changes were less pronounced after mesotherapy. Table 6 contains detailed data.

**TABLE 6:** Comparison of treatments in terms of the effect measured by the change in the level of *Cutibacterium acnes* colonization.

Skin parameter	Measurement area	Treatment group	Min	Max	*M*	Me	SD	*H*	*p*	Post hoc
Percentage of area with fluorescence	T zone	Microneedling (1)	−1.65	4.64	0.48	0.17	1.54	7.399	0.025*	1 > 3
		Exfoliation (2)	−3.70	4.74	−0.48	−0.60	1.94			
		Combined treatment (3)	−3.36	1.21	−0.95	−0.95	1.36			
	Cheeks	Microneedling (1)	−1.31	5.42	0.93	0.39	1.76	15.921	< 0.001***	1 > 2,3
		Exfoliation (2)	−3.74	4.66	−1.18	−1.38	2.03			
		Combined treatment (3)	−2.66	1.60	−0.88	−1.01	1.09			
Number of follicles with fluorescence	T zone	Microneedling (1)	−16.00	46.00	10.55	10.75	15.97	6.918	0.031*	1 > 3
		Exfoliation (2)	−58.00	44.00	0.56	4.13	21.40			
		Combined treatment (3)	−34.25	17.50	−3.22	−3.75	12.67			
	Cheeks	Microneedling (1)	−40.75	51.25	5.76	2.75	18.62	10.979	0.004**	1 > 2,3
		Exfoliation (2)	−52.75	46.50	−9.24	−12.62	21.23			
		Combined treatment (3)	−25.75	13.50	−6.90	−8.00	9.60			
Average fluorescence intensity	T zone	Microneedling (1)	−44.50	4.75	−10.54	−7.50	11.92	30.696	< 0.001***	1 > 2,3
		Exfoliation (2)	−131.00	−37.00	−64.39	−62.50	22.62			
		Combined treatment (3)	−145.50	−6.00	−47.06	−44.37	34.99			
	Cheeks	Microneedling (1)	−66.50	28.75	−12.45	−8.75	19.76	19.056	< 0.001***	1 > 2,3
		Exfoliation (2)	−109.25	39.75	−52.32	−56.62	31.12			
		Combined treatment (3)	−121.25	−7.75	−49.72	−49.25	33.03			

* *p* < 0.05.

** *p* < 0.01.

*** *p* < 0.001.

## 4 Discussion

A comparative analysis showed that among the three interventions studied—microneedle mesotherapy, chemical exfoliation and a combined treatment—the combined procedure had the most comprehensive and beneficial effect on skin parameters, combining improved hydration with a significant reduction in *C. acnes* colonization and a decrease in fluorescence intensity, while maintaining the balance of physiological parameters such as pH, sebum and gloss, although not all skin parameters differed significantly between groups. Both the analysis of skin parameters and fluorescence indicators suggest that combining exfoliation with microneedle mesotherapy may lead to more synergistic effects than using these procedures separately.

A significant increase in skin hydration was observed in all analyzed groups, but the course of changes differed depending on the procedure used. In groups subjected to single methods—exfoliation alone and mesotherapy alone—the improvement was more pronounced only after a full series of six treatments, with a more dynamic effect on the cheeks than on the T‐zone. This is consistent with the available literature, which indicates the gradual action of both alpha hydroxy acids and microneedling, particularly in terms of increasing skin hydration and strengthening its protective barrier [22, 23, 24]. The most uniform and fastest effect was observed in the combined treatment group, where after only three procedures corresponding to exfoliation alone, the level of hydration increased significantly in both the T‐zone and the cheeks. This indicates that it was the mandelic acid exfoliation that was the key stimulus responsible for the early improvement in hydration. This is consistent with the mechanism of action of AHAs, which increase the epidermis' ability to bind water by reducing keratinisation and smoothing the stratum corneum [25]. At the same time, the fact that the level of hydration was maintained after three subsequent microneedle mesotherapy treatments suggests that microneedling may have stabilized the previously achieved effect, probably by influencing the remodeling and regeneration of the skin barrier. This mechanism is confirmed by studies showing that microneedling increases both skin permeability and its regenerative capacity [26].

Significantly different response patterns were observed in terms of skin oiliness. In the group undergoing microneedle mesotherapy, a significant decrease in sebum levels in the T‐zone occurred only after a full series of six treatments, while no statistically significant changes were observed after three treatments. On the cheeks, the reduction in oiliness appeared earlier, after only three treatments, which may be due to lower sebaceous gland activity in this area. The stabilization of sebum levels between the third and sixth treatments suggests that the achieved effect is maintained without further significant changes, which is consistent with earlier reports of the moderate ability of microneedling to modulate sebaceous gland activity [27]. In the chemical exfoliation group, the T‐zone remained resistant to the treatment, and the decrease in sebum production only affected the cheeks and appeared after six treatments. These results are consistent with reports indicating that alpha hydroxy acids, including mandelic acid, have a stronger keratolytic effect than a sebum‐regulating effect, and their impact on sebaceous gland activity is limited, especially in areas with severe seborrhoea [28, 29]. This can be explained by the shallow penetration of AHAs and their predominant effect on the stratum corneum, with little impact on the hair‐sebaceous unit. The most balanced reduction in sebum production was observed in the combined treatment group. Both in the T‐zone and on the cheeks, a significant reduction in sebum levels appeared after six treatments, while the lack of changes after three treatments is consistent with the therapeutic protocol—the first half of the cycle consisted solely of exfoliation, which in itself did not affect the sebum production in the T‐zone. However, the use of microneedle mesotherapy in the second part of the therapy led to a marked reduction in sebum secretion. It is possible that the earlier exfoliation of the epidermis increased the bioavailability of the active substances, which may have enhanced the therapeutic effect [30, 31].

In our study, combined treatments and exfoliation did not cause significant changes in skin pH, indicating their stable safety profile. In contrast, microneedle mesotherapy caused a significant decrease in pH after three treatments, which returned to baseline values after completing a series of six treatments. This phenomenon most likely reflects a temporary skin reaction to microdamage and the healing process, which is consistent with reports that more invasive procedures may temporarily lower pH, while superficial treatments do not cause such deviations [32, 33].

Additionally, analysis of skin physiological parameters showed that neither exfoliation, microneedle mesotherapy, nor the combined treatment caused statistically significant changes in skin gloss. Skin gloss remained unchanged, which may be due to the simultaneous effects of the treatments: pore tightening and sebum reduction, which balanced out potential changes in skin gloss.

The results of the fluorescence parameters showed that the analyzed interventions differed in terms of their effect on the metabolic activity of *Cutibacterium acnes*. The most consistent therapeutic effect was achieved after the combined treatment, which led to a significant reduction in both the area of skin covered by fluorescence and the average fluorescence intensity, and caused a decrease in the number of follicles showing fluorescence on the cheeks. This may indicate a synergistic effect of both treatments, involving both a reduction in keratinisation (exfoliation) and modulation of the hair follicle microenvironment (microneedling) [34]. The main effect of exfoliation was a reduction in the percentage of the area showing fluorescence and the intensity of fluorescence, resulting from the elimination of keratinised epidermis and the reduction of organic substrates for bacteria, which has been widely described in the literature [35]. Microneedling showed a complex effect: despite an increase in the number of fluorescent pores in the T‐zone, the intensity of fluorescence decreased, which may suggest that the procedure primarily affects the metabolic activity of bacteria rather than their number. This is consistent with observations indicating that microneedling modulates skin regeneration processes and alters its microenvironment [36]. When interpreting the results, it is worth emphasizing that contemporary models of acne pathogenesis emphasize the importance of the metabolic activity of *C. acnes* and not just its presence. Porphyrins, which are by‐products of bacterial metabolism, are considered a sensitive marker of the severity of inflammatory processes [37]. The measurements used in the study were based on UVA‐induced fluorescence, in accordance with the principle of UV‐induced red fluorescence (UVRF). This technique is recognized as a reliable tool for assessing the metabolic activity of *C. acnes* and is used, among other things, in the diagnosis of acne [38]. Since the Visiopor device uses the same principle of porphyrin fluorescence emission, its use in the study is fully justified methodologically.

The study has a number of strengths. First of all, three different therapeutic protocols were used, which allowed for a direct comparison of the effects of exfoliation, microneedling, and combination therapy. The use of objective, standardized instrumental measurements increases the reliability of the results obtained and minimizes the influence of subjective assessment. An additional advantage is the multi‐parameter assessment, covering both the physiological properties of the skin and indicators of *C. acnes* bacterial activity, which allowed for a comprehensive analysis of the therapeutic effects. The study also used repeated measurements at three time points, making it possible to track the dynamics of changes.

However, the study also has significant limitations. The small sample size and the fact that it included only women limit the generalisability of the results. Another limitation is that the assessment of *C. acnes* activity was based solely on porphyrin fluorescence measurements, which are a useful but indirect marker of metabolic activity without the ability to distinguish between bacterial numbers, strains, or changes in the skin microbiome structure. Furthermore, the lack of long‐term observation makes it impossible to assess the durability of the effects obtained.

## 5 Conclusion

Each of the treatment protocols presented has its advantages, and therapeutic methods should be selected individually for each patient. The advantages of the treatments include a significant increase in skin hydration, reduction of seborrhoea, and no impact on skin shine. The combination of mandelic acid exfoliation with microneedling has achieved results that are more multidirectional than the effects of each method used separately. After just three treatments, there is a noticeable increase in skin hydration, which is due to the effect of mandelic acid exfoliation, and after completing the full cycle of treatments, there is the strongest reduction in oiliness in both analyzed areas of the face. Fluorescence parameters confirmed the greatest decrease in both the area covered by fluorescence and the intensity of porphyrins, indicating effective modulation of *C. acnes* metabolic activity. This approach allows for the simultaneous normalization of sebaceous gland function, strengthening of the epidermal barrier, and reduction of *C. acnes* microbial activity, which translates into a comprehensive improvement in the condition of oily skin.

## Author Contributions

A.C. contributed to the study concept, recruitment of participants, data collection, analysis and interpretation of results, and drafted the manuscript. E.F., J.B., and D.R. provided critical revision of the manuscript and supervised the study, ensuring scientific and methodological accuracy. All authors approved the final version of the manuscript and agree to be accountable for all aspects of the work.

## Funding

The authors have nothing to report.

## Ethics Statement

All procedures were performed in accordance with the principles of the Declaration of Helsinki, and the study protocol was approved by the Bioethics Committee at the Medical University of Lublin (approval no.: KE_0254/111/04/2023). Each participant was informed in detail about the purpose and course of the study and gave their written, informed consent to participate in the study.

## Consent

Written informed consent was obtained from all patients.

## Conflicts of Interest

The authors declare no conflicts of interest.

## Data Availability

The data presented in this study are available on request from the corresponding author.
